# Effect of chewing hard material on boosting brain antioxidant levels and enhancing cognitive function

**DOI:** 10.3389/fnsys.2024.1489919

**Published:** 2024-11-27

**Authors:** Seungho Kim, Ji-Hye Kim, Hansol Lee, Sung Ho Jang, Ralph Noeske, Changho Choi, Yongmin Chang, Youn-Hee Choi

**Affiliations:** ^1^Department of Medical and Biological Engineering, Kyungpook National University, Daegu, Republic of Korea; ^2^Department of Preventive Dentistry, School of Dentistry, Kyungpook National University, Daegu, Republic of Korea; ^3^Institute for Translational Research in Dentistry, Kyungpook National University, Daegu, Republic of Korea; ^4^Department of Physical Medicine and Rehabilitation, College of Medicine, Yeungnam University, Daegu, Republic of Korea; ^5^GE HealthCare, Munich, Germany; ^6^Department of Radiology and Radiological Sciences, Vanderbilt University Medical Center, Nashville, TN, United States; ^7^Department of Radiology, Kyungpook National University Hospital, Daegu, Republic of Korea; ^8^Department of Molecular Medicine, School of Medicine, Kyungpook National University, Daegu, Republic of Korea

**Keywords:** mastication, hardness, functional magnetic resonance imaging (fMRI), cognitive function, motor function, brain activation

## Abstract

**Introduction:**

Chewing has been reported to enhance cognitive function through the increase in cerebral blood flow. However, the mechanisms linking cerebral blood flow increase to metabolic changes in the brain affecting cognition remain unclear. We hypothesized that glutathione (GSH) plays a pivotal role in these mechanisms. Therefore, this study aimed to investigate changes in brain GSH levels following chewing and their association with cognitive function in healthy young adults.

**Methods:**

A total of 52 university students were recruited, and the Korean version of the Repeatable Battery for the Assessment of Neuropsychological Status was used for the neurocognitive evaluations. Brain GSH levels following chewing gum or wood blocks were measured using MEscher-GArwood Point RESolved Spectroscopy (MEGA-PRESS) sequence, and their relevance to neurocognitive evaluation results was investigated.

**Results:**

Chewing significantly increased brain GSH concentration, particularly in the wood-chewing group compared to the gum-chewing group, as observed in the anterior cingulate cortex. Furthermore, the rise in GSH concentration in the wood-chewing group was positively correlated with memory function.

**Conclusion:**

Chewing moderately hard material elevates brain antioxidant levels such as GSH, potentially influencing cognitive function.

## Introduction

Recent evidence has suggested a potential link between mastication and cognitive function (Lin, 2018; Yokoyama et al., 2017; Onozuka et al., 2003; De Cicco et al., 2016; Fantozzi et al., 2021). This connection may stem from mastication’s ability to influence neuronal metabolism and regional cerebral blood flow across multiple brain areas, including the prefrontal cortex (PFC). For instance, chewing moderately hard food increases cerebral blood flow, while chewing soft and elastic food like gum of varying hardness levels significantly increases brain activity (Suzuki et al., 1994; Onozuka et al., 2002). High levels of blood oxygen have been observed in brain regions with heightened cerebral blood flow, such as the PFC and hippocampus, which are crucial for mastication and cognitive processes like learning and memory (Yokoyama et al., 2017). Clinical studies suggest that individuals experiencing chewing difficulties tend to exhibit lower cognitive function and greater cognitive impairment, whereas activation of cerebral blood appears to positively impact various cognitive functions (Tada and Miura, 2017). However, the precise metabolic changes in the brain associated with cognition following cerebral blood flow increase remain unclear.

Oxidative damage to the brain is believed to play a key role in the decline of cognitive function (Head, 2009; Collin, 2019). Studies on both normal and pathological brains, including those affected by neurodegenerative diseases and aging, have demonstrated higher levels of oxidative damage in aged brains compared to younger ones (Montine et al., 2002). The brain is highly vulnerable to oxidative damage for several reasons. First, human brain consumes approximately 20% of the body’s total oxygen and contains a high amount of polyunsaturated fatty acids, making it susceptible to peroxidation. Second, the accumulation of redox-active metals (such as iron, copper, and zinc) in the brain can catalyze the formation of reactive oxygen species (ROS). Several metabolic processes generate ROS, which can lead to accumulated oxidative damage in human brain (Baierle et al., 2015; Uysal et al., 2018).

To defend against ROS, the antioxidant system consists of several enzymes, such as catalase, superoxide dismutase, glutathione peroxidase, and numerous non-enzymatic endogenous antioxidants such as glutathione (GSH) (Mehta and Gowder, 2015). Among these, GSH is the most crucial antioxidant in the brain for defense against oxidative stress (Meister and Anderson, 1983). In normal adults, GSH concentration typically ranges from 1 to 3 mM (Sanaei Nezhad et al., 2017). Neurons in the brain require high levels of GSH to mitigate oxidative stress, maintain structural integrity, and support cognitive function (Uttara et al., 2009). Magnetic resonance spectroscopy (MRS) has been employed to examine cortical GSH levels. However, measuring GSH with MRS presents challenges due to the low GSH concentration, low signal-to-noise ratio of the brain spectra, and significant spectral overlap between metabolites with varying peak intensities (Lee and Kim, 2019). Consequently, various methods have been proposed to assess GSH concentration *in vivo* within the human brain (Lagopoulos et al., 2013; Mlynárik et al., 2006; Zhao et al., 2006; Mescher et al., 1998). Among these, the MEscher-GArwood Point RESolved Spectroscopy (MEGA-PRESS) sequence has been widely recommend (Rae and Williams, 2007; Terpstra et al., 2006). This spectral editing MRS method is extensively utilized to distinguish and quantify specific metabolites.

Despite the recognition of the crucial role of GSH, how GSH levels in the brain change with mastication remains unexplored. Additionally, the impact of chewing hardness on the correlation between GSH levels and mastication has not been previously studied. In this study, we utilized MEGA-PRESS to measure brain GSH levels in a healthy cohort and investigate their associations with mastication. The effects of chewing hardness on brain’s GSH levels were then examined using materials with different hardness, including gum and wood blocks. The anterior cingulate cortex (ACC), which is pivotal for cognitive control, was selected as the regions of interest. Subsequently, we explored the relationship between brain GSH levels and cognitive function. Our hypotheses were as follows: (1) brain GSH levels correlate with chewing hardness, and (2) chewing hardness influences the relationship between changes in GSH levels and cognitive function.

## Methods

### Participants

The study involved 52 healthy university students from Daegu, Korea. The sample size was determined using G-power software with an effect size (Cohen’s d) of 0.8, significance level of 0.05, and power of 0.8 (Poldrack et al., 2011). Participants were voluntarily participated in the study through recruitment notice, and individuals with neurological disease, psychiatric illness, temporomandibular joint disorders, or contraindications to magnetic resonance imaging were excluded. The participants were divided into two groups, including the gum-chewing (*n* = 27) and wood-chewing (*n* = 25) group, ensuring similarity in age, sex, and education level between the groups. Sociodemographic information, including age, sex, education, and general health status, were collected via a self-reported questionnaire. This study was approved by the Institutional Review Board of Kyungpook National University Dental Hospital (KNUDH-2021-06-09-02), and all participants provided informed consent prior to participation.

### Neuropsychological measurement

The Korean Repeatable Battery for the Assessment of Neuropsychological State (K-RBANS) was administered by a clinical psychologist. The K-RBANS neuropsychological test assesses five indices within 30-min timeframe, covering immediate and delayed memory, visuospatial capacity, verbal ability, and attention (Park et al., 2021). The results of the K-RBANS are reported as scores for the five indices, scores for the 12 subtests, and a total scale score. This format enables a comprehensive evaluation of the participant’s cognitive function as well as an assessment of specific cognitive domains.

### Chewing task

Participants were positioned supine with their heads stabilized using a fixation device and instructed to chew either paraffin wax gum (CAT 21 Buf Test, Morita Co., Japan) or a wood stick (Tongue Depressors, Daehan medical, Korea) for 5 min. To ensure consistent movement, participants chewed on the right molar region at a frequency of 1 Hz, alternating between 30 s of chewing and 30 s of rest.

### MRS data measurement

All MRS data were acquired using a 3.0 T GE SIGNA Architect MR scanner with a 48-channel head coil before (MAS_before) and after mastication (MAS_after). MRS data were obtained using a GSH-edited MEGA-PRESS sequence on a single voxel of 2 × 2 × 2 cm^3^ located at the ACC (repetition time = 2,000 ms, echo time = 79 ms, number of averaging = 128, total sampling points = 4,096, and spectral width = 5 kHz). A 20-ms Gaussian 180° RF pulse (bandwidth = 60.4 Hz), serving as an editing pulse, was applied before and after the second 180° slice-selective RF pulse at 7 ppm in the editing-off (Edit-OFF) scan and at 4.56 ppm coupled with GSH spin resonance at 2.95 ppm in the editing-on (Edit-ON) scan within the MEGA-PRESS sequence. Individual Edit-ON and Edit-OFF resonance spectra were alternately acquired. Vender-supplied tools were used for B_1_ calibration, water suppression (water linewidth = 8.1 ± 1.7 Hz for MAS_before, 8.0 ± 1.5 Hz for MAS_after), and B_0_ shim (zero, first, and second order). At the beginning of each scan, an unsuppressed reference water signal was acquired for eddy-current correction and multichannel combination. Foam pads were utilized inside the head coil to minimize head movement.

### MRS data processing

**Figure 1:**
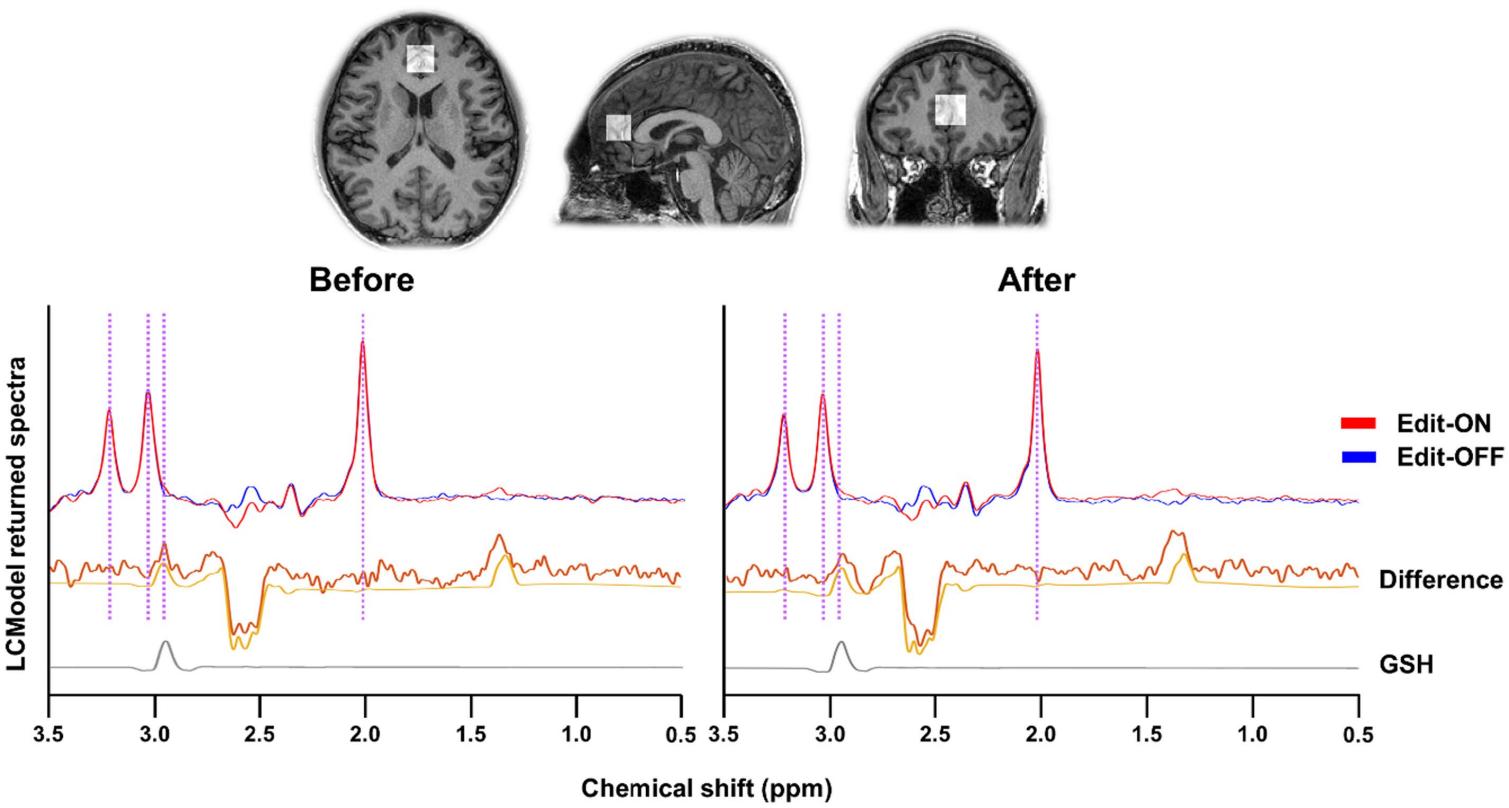
The representative GSH-edited spectra of mastication before and after from anterior cingulate cortex (ACC) by MEGA-PRESS method. Editing on (Red line) and off (Blue line) spectra were aligned and differenced. The difference spectra and spectral fitting results were described as the yellow thick and thin lines, respectively. Because GSH signal in the MR spectra is overlapped with other metabolite signals, the spectral editing technique with edit-on and edit-off MRS sequences was employed to give only GSH signal as shown in the bottom (gray line). The purple vertical lines indicate the peaks of NAA (2.01 ppm), GSH (2.95 ppm), Cr (3.03 ppm), Cho (3.21 ppm).

Individual MR spectra were obtained using apodization with a 1.5-Hz exponential function and underwent frequency and phase-shift correction. The Edit-ON and Edit-OFF spectra were aligned by minimizing the difference signals of N-acetyl-aspartate (NAA) at 2.01 ppm, choline at 3.21 ppm, and creatine at 3.03 ppm to determine the GSH-edited difference spectra. To estimate the levels of metabolites in the GSH-edited difference and Edit-OFF spectra, the LCModel^1^ was applied. The basis set for spectral fitting of GSH-edited difference spectra included GSH, NAA, N-acetylaspartateglutamate (NAAG), aspartate, lactate, myo-inositol (MI), phosphocholine (PCh), glycerophosphocholine (GPC), threonine, phosphoethanolamine, and glucose. The basis set for spectral fitting of Edit-OFF spectra additionally included glutamine, glutamate, creatine (Cr), phosphocreatine (PCr), glycine, alanine, acetate, ethanolamine, ascorbic acid, taurine, and serine, with LCModel built-in macromolecular and lipid basis signals. The metabolite basis set was determined using a simulation tool incorporating the MEGA-PRESS sequence diagram, spectral and slice-selective RF, and gradient pulse strength from a published simulation algorithm (Choi et al., 2012). After GSH estimates were normalized to the unsuppressed water, millimolar concentrations were calculated by assuming a mean total creatine concentration of 8 mM (Mekle et al., 2009; Tkac et al., 2009). The quality of spectral fitting was assessed using the Cramér–Rao lower bounds (CRLB) as the error estimate of the concentration measurement, with each GSH CRLB result being <20%, indicating reliable measurement (Mandal et al., 2015; Mandal et al., 2023). The fitting of GSH using the MEGA-PRESS sequence is illustrated in Figure 1.

### Statistical analysis

The Statistical Package for Social Sciences (SPSS 25; https://www.ibm.com/kr-ko/products/spss-statistics) was used for all statistical analyses. An independent samples *t*-test was conducted to compare the neuropsychological scores and GSH levels between participants in the gum-and wood-chewing groups. A paired *t*-test was performed to investigate changes in GSH levels before and after mastication. Pearson’s coefficients were calculated to examine correlations between GSH levels in ACC and neuropsychological scores.

## Results

### Demographic characteristics and neuropsychological analysis

The gum-chewing group consisted of 13 men and 14 women, and the wood-chewing group consisted of 11 men and 14 women. The average age of all participants was 21.81 ± 1.56 years, with the gum-chewing group averaging 22.00 ± 1.66 years and the wood-chewing group averaging 21.60 ± 1.44 years. Participants had an average education level of 14.63 ± 1.14 years, with the gum-chewing group averaging 14.74 ± 1.06 years and the wood-chewing group averaging 14.52 ± 1.23 years. No significant difference was found in the sociodemographic characteristics between the two groups (*p* > 0.05) (data not shown).

No statistically significant difference was observed in the K-RBANS assessment scores between the gum-and wood-chewing groups (*p* > 0.05) (Table 1).

**Table 1:** Comparison of neuropsychological evaluation by K-RBANS scores of the subjects.

	Total (*N* = 52)	Gum (*N* = 27)	Wood (*N* = 25)	*p*-value
Index				
Immediate memory	92.63 ± 12.31	91.30 ± 13.66	94.08 ± 10.76	0.51
Visuospatial/constructional	100.58 ± 13.86	99.96 ± 13.80	101.24 ± 14.18	0.73
Language	92.02 ± 17.95	90.70 ± 16.30	93.44 ± 19.82	0.61
Attention	105.02 ± 16.12	103.74 ± 17.98	106.40 ± 14.07	0.74
Delayed memory	94.15 ± 16.31	92.11 ± 16.17	96.36 ± 16.50	0.39
Total scale	96.63 ± 14.81	94.59 ± 15.32	98.84 ± 14.22	0.36
Subtest				
List learning	30.46 ± 3.13	30.11 ± 3.70	30.84 ± 2.39	0.50
Story memory	19.08 ± 2.56	18.89 ± 2.46	19.28 ± 2.70	0.52
Figure copy	19.71 ± 0.87	19.56 ± 1.12	19.88 ± 0.44	0.25
Line orientation	17.81 ± 2.13	17.96 ± 1.89	17.64 ± 2.40	0.75
Picture naming	8.92 ± 1.03	8.85 ± 1.10	9.00 ± 0.96	0.69
Semantic fluency	20.35 ± 4.54	20.15 ± 4.26	20.56 ± 4.90	0.65
Digit span	14.38 ± 2.27	14.15 ± 2.32	14.64 ± 2.23	0.26
Coding	71.35 ± 8.85	71.52 ± 10.53	71.16 ± 6.79	0.90
List recall	7.67 ± 1.64	7.59 ± 1.42	7.76 ± 1.88	0.56
List recognition	19.73 ± 0.60	19.70 ± 0.54	19.76 ± 0.66	0.42
Story recall	10.35 ± 1.28	10.22 ± 1.42	10.48 ± 1.12	0.60
Figure recall	16.17 ± 3.57	15.85 ± 4.04	16.52 ± 3.03	0.77

Values are expressed as mean ± standard deviation. *p*-values from group comparisons.

### Metabolites quantification by MRS using MEGA-PRESS

**Figure 2:**
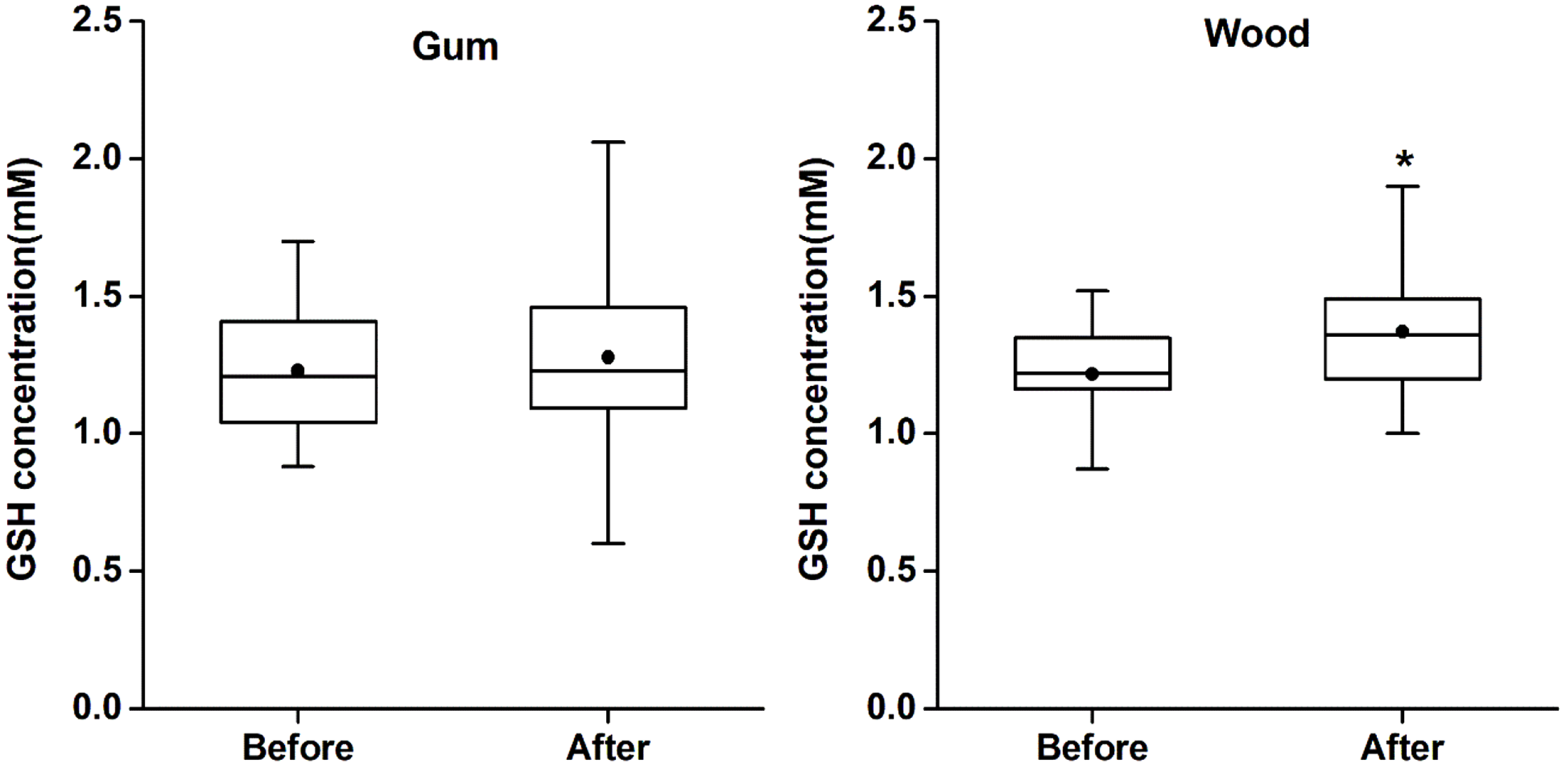
The changes of GSH concentrations in each group. The GSH concentrations (Cramér-Rao lower bound) were 1.23 mM (9%), 1.28 mM (8%) in the gum group, and 1.22 mM (8%), 1.37 mM (8%) in the wood group in mastication before and after, respectively. (^*^p < 0.01). The gum group showed no significant change before and after gum chewing but the wood group showed statistically significant increase in GSH concentration after wood chewing compared to GSH concentration before wood chewing.

GSH levels in ACC (mean ± SD and CRLB) were 1.23 ± 0.24 mM (9% ± 2%) and 1.22 ± 0.18 mM (8% ± 2%) for MAS_before and 1.28 ± 0.33 mM (8% ± 3%) and 1.37 ± 0.23 mM (8% ± 2%) for MAS_after in the gum-and wood-chewing groups, respectively. According to a paired *t*-test, GSH levels in MAS_after were significantly increased compared to MAS_before in the wood-chewing group (*t* = 2.97, *p* = 0.007, effect size = 0.81). However, no significant increase in GSH level was observed in the gum-chewing group (*t* = 0.68, *p* = 0.505, effect size = 0.1) (Figure 2). In an independent samples *t*-test (*p* = 0.237, effect size = 0.33), there was no significant difference observed between the groups in terms of changes in GSH levels. Additionally, the concentration for other metabolites estimated from Edit-off spectra, such as tNAA (total NAA; NAA + NAAG), tCr (total creatine; Cr + PCr), tCho (total choline; PCh + GPC, and MI), were not different among the two groups. The metabolite concentration changes between MAS_before and MAS_after were also insignificant in each group. These results were provided in Supplementary Figure S1.

### Relationship between GSH levels and neuropsychological measurements

**Figure 3:**
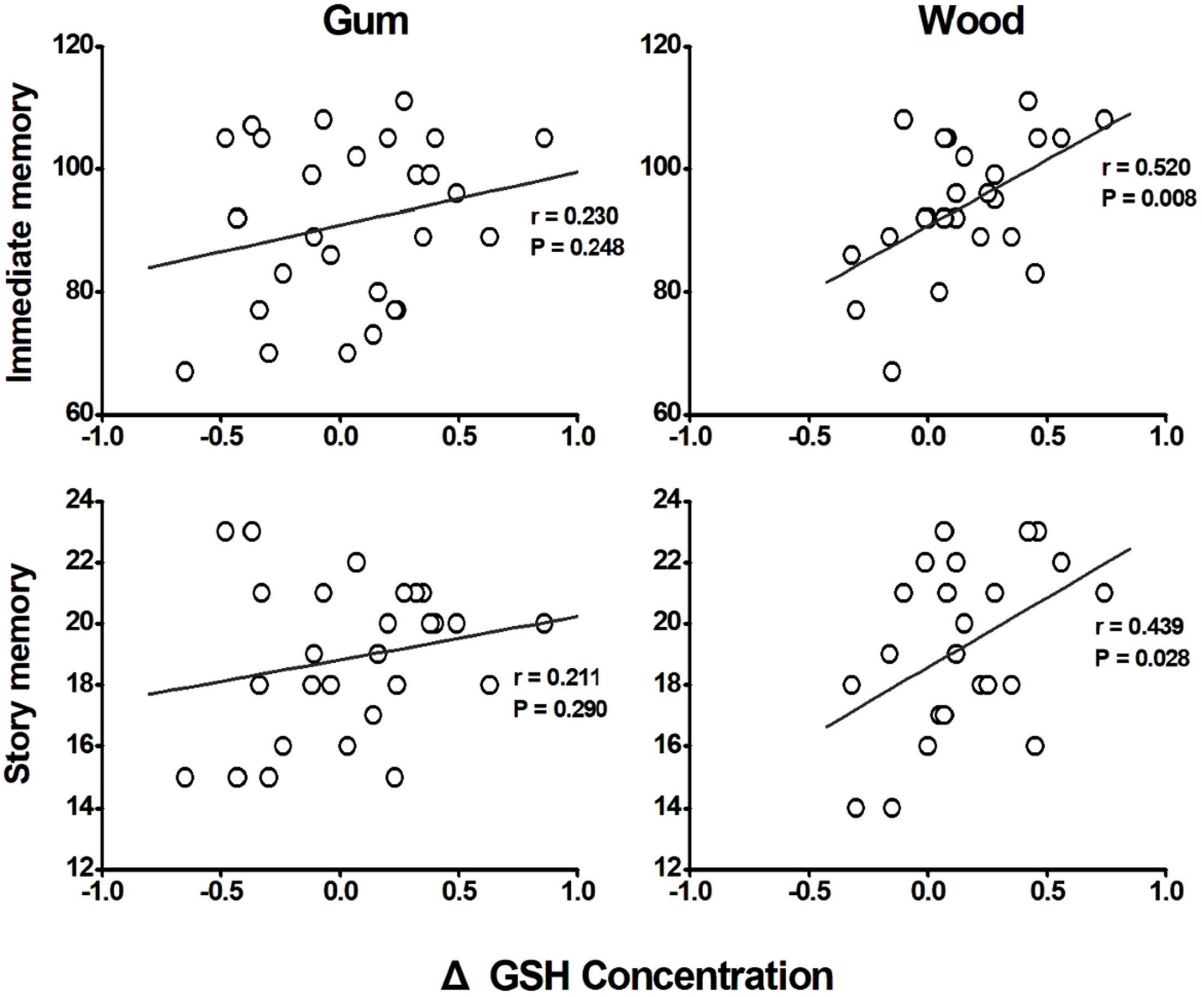
Correlation between the changes of GSH concentrations in anterior cingulate cortex (ACC) and cognitive functions. Both correlations with immediate memory and story memory as cognitive function measure revealed that the gum chewing group did not show any significant correlations between the changes of GSH concentrations and immediate memory and story memory (*p* = 0.248 and *p* = 0.290 respectively). The wood chewing group however showed the significant positive correlations between the changes of GSH concentrations and immediate memory and story memory (*p* = 0.008 and *p* = 0.028 respectively). These results therefore suggest that the increase of GSH concentration by wood chewing increase the cognitive functioning in wood chewing group.

In the wood-chewing group, changes in GSH concentrations demonstrated a positive correlation with immediate memory (*r* = 0.520, *p* = 0.008) and story memory (*r* = 0.439, *p* = 0.028) scores (Figure 3). However, no significant correlations were observed in the gum-chewing group.

## Discussion

In the current study, GSH concentration measured by MRS was significantly higher in the ACC in the wood-chewing group compared to that in the gum-chewing group. Furthermore, the increase in GSH in the wood-chewing group was positively correlated with memory function items in neuropsychological measurements. To the best of our knowledge, this is the first report indicating that mastication can alter the level of antioxidants in the human brain, and that an increase in brain antioxidant levels is associated with cognitive function.

The results from the current study revealed that chewing moderately hard material leads to an increase in the GSH concentration in the brain. GSH is present in almost all cell types and is involved in numerous physiological functions, including detoxication of xenobiotics, intracellular redox homeostasis, cysteine carrier/storage, cell signaling, protein folding and function, gene expression, cell differentiation/proliferation, immune response, and antiviral (Cooper and Kristal, 1997; Pastore et al., 2003). Additionally, GSH acts as a “master antioxidant” across all tissues, particularly in the brain where the GSH plays a crucial role in antioxidant defense (Dringen, 2000). While low levels of ROS are necessary for maintaining normal cellular function, their accumulation leads to oxidative stress, resulting in lipid peroxidation, protein oxidation, and DNA damage that ultimately impair cellular function. The brain is especially vulnerable to oxidative stress. Therefore, the finding that chewing hard materials increases GSH concentration is intriguing and significant because it suggests that mastication may help protect the brain from oxidative stress. Based on these results, we hypothesized that the increase of GSH, brain antioxidant, by chewing hard material may effectively remove the reactive oxygen species in the brain and thus stimulate brain cells to improve the brain cognitive function. Furthermore, this finding may be particularly relevant for elderly individuals, as GSH deficiency contributes to oxidative stress, which is implicated in aging and the pathogenesis of many neurodegenerative diseases (Jenner, 1994; Jenner, 2003; Maher, 2005). An MRS study using MEGA-PRESS sequence provided *in vivo* evidence that the patients with Alzheimer’s disease exhibit reduced GSH concentrations in the frontal cortex compared to age-matched healthy controls (Mandal et al., 2015).

While the findings in this study strongly suggest that chewing relatively hard material is closely associated with an increase in GSH concentration in the brain, the exact cause of this GSH alteration remains to be elucidated. It is possible that the increase in GSH simply reflects an increase in cerebral blood flow (CBF). Several studies have shown that chewing moderately hard food leads to an increase in CBF, and chewing gum with varying degrees of hardness significantly enhances brain activity (Suzuki et al., 1994; Onozuka et al., 2002). Increased CBF has been linked to improved cognitive functions. According to previous reports, it is tempting to hypothesize that the increase CBF may supply sufficient nutrients to the brain to stimulate GSH synthesis. The correlation observed in the current study between the increase in GSH and cognitive measures found seems to support our hypothesis that cognitive function improves as GSH concentration increases.

The findings of the present study suggest important implications for a therapeutic strategies aimed at increasing neuronal GSH levels in the brain. While elevating brain GSH levels holds promises as a treatment for GSH-related neurodegenerative or neuropsychiatric diseases (Gilgun-Sherki et al., 2004; Pittenger et al., 2011), there are currently no therapeutic drugs available for this purpose. Orally administered GSH is rapidly degraded in the gut, and intravenously administered GSH is quickly oxidized to GSSG in the bloodstream with a half-life of 2–3 min (Ammon et al., 1986; Wendel and Cikryt, 1980). Therefore, the direct administration of GSH does not appear promising for treating GSH-related neurodegenerative or neuropsychiatric diseases. On the other hand, the current study demonstrated that chewing moderately hard materials such as wood blocks can increase brain GSH levels without degradation in the gut or oxidization in the blood. Hence, mastication, especially with moderately hard material, may offer a practical approach to enhancing GSH levels in the brain and is beneficial for maintaining or bolstering antioxidant defense in the brain.

This study has several limitations that should be addressed. Firstly, the participants in the present study were aged 20–30 years and were selected using convenience sampling. Due to this narrow age range, the findings need further validations across broader age groups, including both younger and older individuals. Secondly, the study population was limited in size, necessitating caution when generalizing the findings to a larger populations. Thirdly, our study focused specifically on the ACC, and thus, the results may not be applicable to other brain regions. Future research could explore GSH levels across a broader range of brain regions to gain a more comprehensive understanding of region-specific changes in GSH in response to chewing hardness. Fourthly, while our study limited chewing time to 5 min using relatively soft gum, longer chewing durations are possible with softer gums, potentially affecting brain activity. Moreover, chewing gum and wood can be differed not only in hardness but also in various other characteristics such as texture of the materials. Investigating changes in GSH concentrations that reflect real-world chewing scenarios will be necessary in future research. Fifthly, we were unable to objectively measure chewing hardness. Further research is needed to quantify chewing hardness and determine the optimal chewing force that can enhance cognitive function. These limitations highlight avenues for future studies to further elucidate the effects of chewing on GSH levels in the brain under diverse conditions and across different populations.

In summary, this study yielded two major findings. Firstly, the wood-chewing group experienced stimulated of brain GSH synthesis, leading to increased GSH levels in the brain. Secondly, correlation analysis indicated that the higher GSH levels in the wood-chewing group were associated with improved scores in cognitive measures. Since there are currently no drugs or established practices for boosting brain GSH levels, our findings suggest that chewing moderately hard material could serve as an effective practice for increasing GSH levels in the brain. Based on these results, consuming harder foods might prove more effective in enhancing brain antioxidant defenses through elevated GSH levels.

## Data Availability

The raw data supporting the conclusions of this article will be made available by the authors, without undue reservation.

## Footnotes

^1^ http://s-provencher.com/lcmodel.shtml
