# Supplementary material for: Effect of chewing hard material on boosting brain antioxidant levels and enhancing cognitive function
Source: Front Syst Neurosci. 2024 Nov 27;18:1489919. doi: 10.3389/fnsys.2024.1489919 (PMC11632103; doi:10.3389/fnsys.2024.1489919)
Supplement: Supplementary file 1 [file Table_1.DOCX]

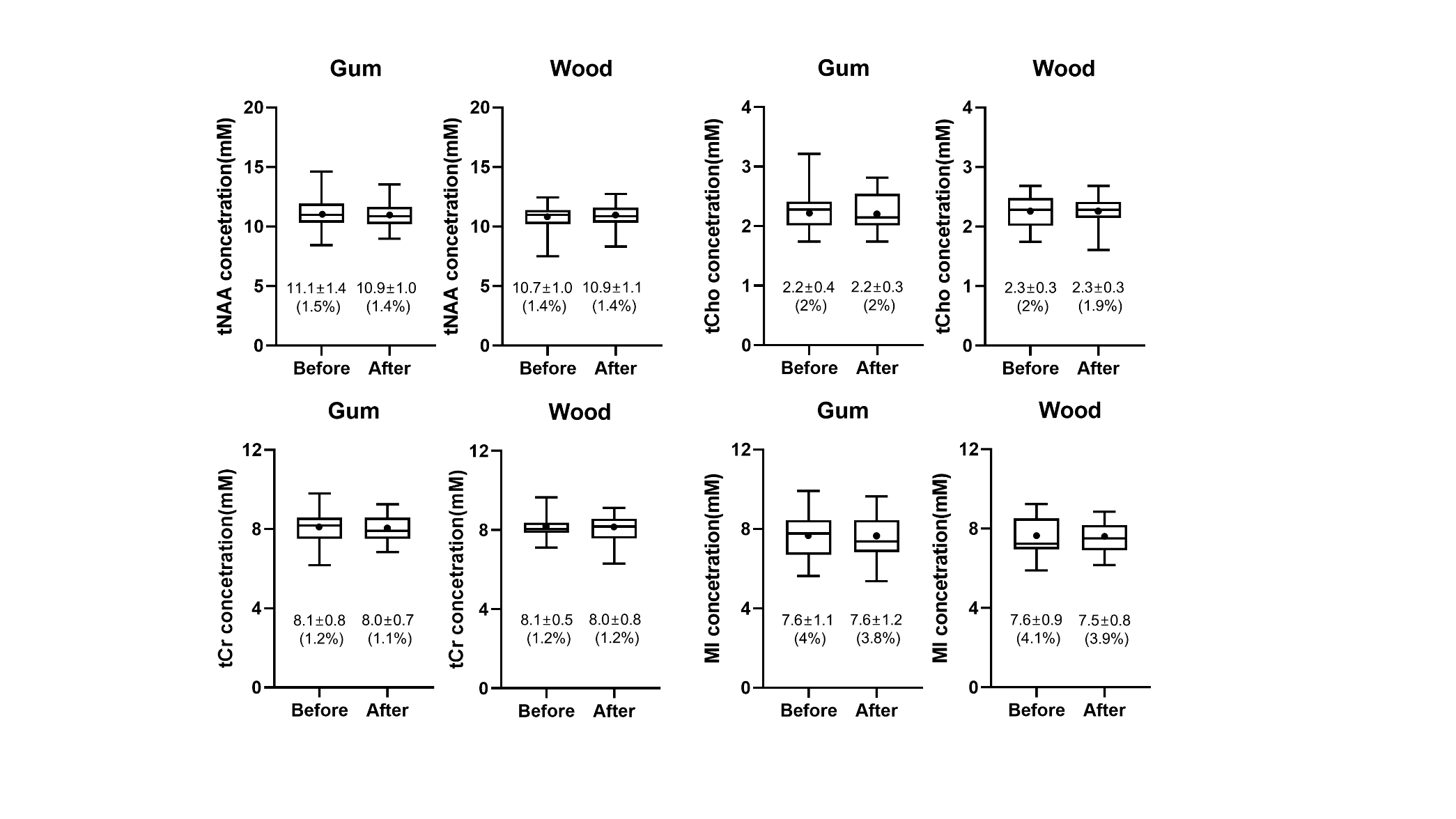


**Supplementary figure 1. Metabolites concentration estimated from editing off spectra.** The concentration for metabolites estimated from Edit-off spectra, such as tNAA (total NAA; NAA + NAAG), tCr (total creatine; Cr + PCr), tCho (total choline; PCh + GPC), and MI, were not different between the gum-chewing and wood-chewing groups. The metabolite concentration changes before and after mastication were also insignificant in each group.
